# Clinical outcomes and long-term efficacy of high tibial osteotomy in treating knee instability: An updated systematic review

**DOI:** 10.1051/sicotj/2024061

**Published:** 2025-01-23

**Authors:** Edi Mustamsir, Aulia Pandu Aji, Ahmad Abdilla Adiwangsa, Azfar Ahnaf Akmalizzan

**Affiliations:** 1 Orthopaedics and Traumatology Department, Faculty of Medicine, Brawijaya University – Saiful Anwar General Hospital Malang 65142 East Java Indonesia; 2 General Practitioner Malang 65142 East Java Indonesia; 3 Bhayangkara Hospital Kediri 65142 East Java Indonesia; 4 Ngudi Waluyo General Hospital Blitar 65142 East Java Indonesia

**Keywords:** HTO, Outcomes, Instability, Tibial, Slope

## Abstract

*Introduction*: Knee joint stability is influenced by force distribution and ligament structures. High Tibial Osteotomy (HTO) treats knee deformities and redistributes load, reducing further invasive procedures. High Tibial Osteotomy (HTO) is a well-established procedure for addressing knee instability, particularly in cases involving ligament deficiencies such as ACL and PCL insufficiencies. This systematic review aims to evaluate the clinical outcomes and long-term efficacy of HTO in improving knee stability and function. *Methods*: A systematic literature search was conducted using Cochrane Central, PubMed, MEDLINE, and ProQuest databases for studies published between 2000 and June 2024. Eligible studies included human subjects with at least six months of follow-up and focused on HTO for knee instability. Exclusion criteria included animal studies, non-knee joint studies, and reviews. Data on patient demographics, follow-up duration, subjective and objective outcomes, and complications were extracted. *Results*: Out of 536 studies identified, 11 met the inclusion criteria, encompassing 303 patients. Combining HTO with ACL or PCL reconstruction significantly improved both subjective instability and objective measures, including Lachman and Pivot Shift test grades. Patient satisfaction was high, and functional scores such as Lysholm and Tegner improved markedly. The incidence of complications was low, with minor issues such as infections and delayed union, and no reported graft failures. *Conclusion*: HTO, particularly when combined with ligament reconstruction, effectively treats knee instability due to ACL or PCL deficiency. The procedure demonstrates strong mid- to long-term outcomes, high patient satisfaction, and a low rate of complications. It remains a viable option for patients with knee instability.

## Introduction

Knee joint stability is generally influenced by the distribution of force in the coronal plane, sagittal plane and rotational plane. This joint consists of multiple intra and extra-articular ligamentous structures around it. Acquired or congenital deformities of the lower extremities altered knee biomechanics, eventually impacting the force transmitted through the ligament [1]. Based on the findings, we can determine that the transmitted force can be modified for redistribution.

Knee osteotomy is an effective procedure to treat different deformities and redistribute the load at the joint level, reducing the risk of wear and, consequently, the need for invasive procedures. The commonly applied procedure is High tibial osteotomy (HTO). It stands out as a valuable intervention for various acute and chronic knee instability. The expanding role of HTO transcends knee osteoarthritis to encompass acute or chronic knee instabilities, including ligament reconstructions and failures. This approach is held by adjusting the lower extremities weight-bearing line (WBL) to achieve desired outcome. Alignment correction in knees with ligament deficiency is critical to prevent further compartment degradation, safeguard grafts, and reinstate stability. The existing literature on HTO and knee instability presents diverse viewpoints on indications, timing, and outcomes, with ongoing studies aiming to assess HTO’s impact comprehensively. Two kinds of high tibial osteotomy are usually applied in practice. To this time, Open-wedge HTO (OWHTO) is commonly employed, with OWHTO gaining popularity for its less invasive nature, simplicity, and ability to fine-tune alignment intraoperatively. MOWHTO demonstrates precise deformity correction advantages and potential adaptability to total knee arthroplasty without requiring fibula osteotomy, making it a preferred choice for active, younger patients with medial compartment knee instability. Effective preoperative planning in OWHTO involves delineating the target WBL passing through a specific tibial plateau point on standing whole-leg radiographs [2, 3]. Older study was unable to identify whether HTO alone is better than HTO with simultaneous ligament reconstruction. By conducting this study, we aim to clarify HTO outcomes with the addition of latest studies for better decision-making and complication prevention, also to measure improvement of stability as well as satisfication and return to sport.

This systematic review aims to provide clear information of the outcome from the utilization of high tibial osteotomy in patients with knee instability, both in acute and chronic cases to contribute high-precision decision making in the following procedure and prevent further complication.

## Methods

This study was registered in The International Prospective Register of Systematic Reviews (PROSPERO) database with the registration number CRD42024562555 adhered to the 2020 PRISMA guidelines [4]. A systematic review of the literature on the treatment of knee ligamentous instability with high tibial osteotomy (HTO) was conducted using the Cochrane Central Register of Controlled Trials, PubMed, MEDLINE, and ProQuest databases to the present, with searches performed in June 2024.

The literature search strategy included: search 1: (“HTO” OR “High tibial osteotomy” OR “PTO” OR “Proximal Tibial Osteotomy”) AND (unstable OR instability OR laxity OR subluxation OR “tibial slope” OR “knee malalignment” OR “knee alignment” OR “chronic posterolateral” OR “revision knee surgery”); search 2: “tibia” AND “osteotomy” AND (unstable OR instability OR laxity OR subluxation). Inclusion criteria were high or proximal tibial osteotomy to treat knee joint instability, a minimum half-year follow-up with outcome reports, English language, and human studies. We have considered retrospective studies as eligible for inclusion. Exclusion criteria included animal studies, basic science studies, cadaveric studies, editorials, reviews, expert opinions, surveys, special topics, letters to the editor, and correspondence. Additionally, studies on joints other than the knee were excluded.

Four investigators (EM, AP, AAA, AAA) independently reviewed the abstracts of all identified articles. Full-text articles were obtained for review if necessary to apply the inclusion and exclusion criteria. Additionally, all references from the included studies were reviewed to ensure no relevant articles were missed. Inclusion and exclusion criteria were sequentially applied to identify relevant articles, as shown in the PRISMA flowchart.

Data Collection Following the classification by Wright et al. [5], the level of evidence was assigned [4]. Information was collected from the abstracts of the included studies. Pre- and postoperative instability was recorded as subjective instability, Lachman test, and pivot shift test. Additionally, patient demographics, follow-up, patient satisfaction, subjective outcomes, return to sport, and complications were recorded. For continuous variables (e.g., age, timing, follow-up, outcome scores), the mean and range were collected if reported. Data was recorded into a custom Microsoft Excel spreadsheet (Microsoft Corp) using a modified information extraction table.

Bias there can be inherent selection and performance bias in level 3 and level 4 studies due to the lack of randomization and prospective comparative control groups, especially in populations characterized by injury heterogeneity. Selected studies were reviewed to ensure that authors minimized bias while recognizing the constraints present in such studies (Tables 1–3).

**Table 1:** Knee instability.

Author, year	Country	Mean	No of patients (M/F)	Age (mean)	BMI	Ligament instability	Procedure	Subjective instability	Lachman	Pivot shift
		Follow-up, y								
Badhe, 2002 [6]	United Kingdom	2.8 years (mean)	14 (9/5)	N.A	N.A	5/14 ACL (DV);	HTO + ACLR/PCLR/PLRI	Pre-op: 100%		
						2/14 PCL + PLRI (TV);	5/14 Single stage CWTO + ACLR;	Post-op: 0%		
						2/14 ACL + PLRI (TV);	1/14 Single stage HTO + PCL + PLRI;			
						1/14 ACL + PCL + PLRI (TV)	1/14 Single stage HTO + ACL + PLRI;			
						1/14 PLRI (TV)	1/14 ACL + PLRI followed by HTO;			
						2/14 PCL + PLRI (TV)	1/14 OWHTO + PCL + PLRI;			
						1/14 PLRI (TV)	1/14 HTO + PCL + PLRI;			
							1/14 HTO + PLRI;			
							2/14 OWHTO;			
							1/14 HTO			
Jin, 2018 [7]	South Korea	5.2 y	24 (20/4)	40.2 years (range: 29–52 years)	N.A	24/24 ACL + Medial compartment OA	HTO + ACLR		Grade 0: 0.14	Grade 0: 0.15
									Grade I: 3.7	Grade I: 9.5
									Grade II: 14.3	Grade II: 11.4
									Grade III: 7.0	Grade III: 4.0
									(pre-op, final follow-up)	(pre-op, final follow-up)
									*p* < 0.001	*p* < 0.001
Vaishya, 2016 [8]	India	1.3 y	40 (27/13)	37.3 years	N.A	40/40 ACL + OA	MOWHTO + ACLR	Pre-op: 100%		
						Cause of injury: Sport 85%, domestic injury 15%		Post-op: 0%		
Mehl, 2016 [9]	Germany	5.8 y	52 (36/16)	37.95 years	26.75 kg	52/52 ACL + OA varus	26/52 HTO	Pre-op: 100% Post-op: 0%		
							26/52 HTO + ACLR	Arthroscopy confirmed		
Sonnery-Cottet et al. 2014 [10]	France	2.6 y	5 (4/1)	24 years	N.A	5/5 ACL	5/5 One Stage ACLR(Revision) + ACW-HTO with detachment of ATT and			Grade 0: 0.4
							Patellar tendon			Grade I: 1.1
										Grade II: 3.0
										Grade III: 1.0
										(pre-op, final follow-up)
Song et al. 2020 [11]	China	2.8 y	18 (16/2)	29.4 y	N.A	18/18 ACL	18/18 One Stage ACLR (Primary) + ACW-HTO			Grade 0: 0.18
							Without detachment of ATT and patellar tendon			Grade I: 0.0
										Grade II: 15.3
										Grade III: 3.0
										(pre-op, final follow-up)
Akoto et al. 2020 [12]	Germany	2.5 y	20 (14/6)	27.8 ± 8.6 years	N.A	20/20 ACL	20/20 Two Stages ACLR(Revision) + ACW-HTO with detachment of ATT and patellar tendon			Grade 0: 0.20
										Grade I: 0.0
										Grade II: 0.0
										Grade III: 20.0
										(pre-op, final follow-up)
Rozinthe et al. 2022 [13]	France	9.9 y	8 (5/3)	N.A	N.A	8/8 ACL	8/8 One Stage ACLR (Revision) + ACW-HTO without detachment of ATT and patellar tendon	Pre-op: 100% Post-op: 0%		
Weiler et al. 2022 [14]	Germany	0.5 y	76 (47/29)	32.2 years (range 17–57 years)	N.A	76/76 ACL	76/76 Two Stage ACLR(Primary) + ACW-HTO or MOW-HTO without detachment of ATT and patellar tendon	Pre-op: 100% Post-op: 0%		
Bonin et al. 2004 [15]	France	12 y	29	30 (range 18–41 years)	N.A	29/29 ACL + OA	29/29 One Stage ACLR + HTO	Pre-op, mean 10.6 (Grade III); Post-op, mean 8.1 (Grade II)	–	–
Schneider et al. 2020 [16]	France	10 ± 5.2 y	35 (24/11)	39 ± 9 years	N.A	35/35 ACL	Combined ACL reconstruction (bone-patellar tendon-bone graft) and MOW HTO	Mean IKDC score of 71.8 ± 14.9, Lysholm score of 82 ± 14.1	Pre-op: Positive in 94%, Post-op: Normal in 63%, Firm end in 31%, Soft end in 6%	Pre-op: Abnormal in 89%, Post-op: Normal in 74%, Glide in 20%, Clunk in 6%

*OWHTO* open-wedge tibial osteotomy, *CWTO* closed-wedge lateral tibial osteotomy, *MOWHTO* medial open wedge high tibial osteotomy, *ACW-HTO* anterior closed-wedge high tibial osteotomy, *PCL* posterior cruciate ligament, *PLRI* postero-lateral rotatory instability, *ACL* anterior cruciate ligament, *DV* double varus, *TV* triple varus, *ATT* anterior tibial translation*.*

**Table 2:** Pre-operative and post-operative value of satisfaction, lysholm score, cincinnati knee score, tagner activity score, mechanical axis, IKDC, posterior tibial slope and return to sport/activities.

Author, year	Satisfaction	Lysholm score		Cincinnati knee score		Tegner activity score		Mechanical axis (in degree)		Subjective IKDC questionnaire		Posterior tibial slope		Return to sport/activities
	Post-op	Pre-op (Mean ± SD /(range))	Post-op (Mean ± SD/(range))	Pre-op	Post-op	Pre-op (Mean ± SD/(range))	Post-op (Mean ± SD/(range))	Pre-op (Mean ± SD/(range))	Post-op (Mean ± SD/(range))	Pre-op (Mean ± SD/(range))	Post-op (Mean ± SD/(range))	Pre-op (Mean ± SD/(range))	Post-op (Mean ± SD/(range))	*N* (%)
Badhe, 2002 [6]				74	53									13 (93%) able to participate light recreational activity
Jin, 2018 [11]		58.5 ± 12.0	94.0 ± 5.9			5.3 ± 0.9	4.0 ± 1.1	7.0 ± 2.3	−1.2 ± 1.4			9.1 ± 1.4	10.2 ± 2.3	
Vaishya, 2016 [7]											87.5 (range 60–100)			87.5% participate in preoperative sport; 91% better quality of life; 92.5% return to the same occupation pre-op
Mehl, 2016 [8]	48 (92%) satisfy		Group 1: 69.4 (SD 15.7)						Group 1: 180.4 (SD 3.3)		Group 1: 64.8 (SD 13)			
	3 (6%) moderately satisfied		Group 2 78.39 (SD 16.4)						Group 2: 182.1 (SD 2.1)		Group 2: 74 (SD 15.6)			
	1 (2%) unsatisfied													
Sonnery-Cottet et al. 2014 [12]		46.2 (26–69)	87.8 (60–100)			7.4 (5–9)	7.2 (5–9)			39.5 (21.8– 64–4)	79.1 (48.3–98.9)	13.6° (13°–14°)	9.2° (8°–10°)	4 (80%)
Song et al. 2020 [13]		46.5 (34–58)	89.5 (78–94)			5.7 (4–6)	7.3 (6–8)					18.5° (17°–20°)	8.1° (7°–9°)	18 (100%)
Akoto et al. 2020 [14]		49.9 ± 21 (0–70)	90.9 ± 6.4 (76–100)			2.9 ± 1.5 (0–5)	6.1 ± 0.9 (5–8)				87.4 ± 5.9 (75.9–100)	15.3° ± 11.6°	8.9° ± 11.1°	13 (65%)
Rozinthe et al. 2022 [9]		38.4 ± 16.4 (24–80)	84.5 ± 11.9 (59–95)							44.1 ± 16.1 (23–75)	82.9 ± 12.1 (61–98)	13.2° ± 2.6° (10°–18°)	4.4° ± 2.3° (2°–8°)	N/A
Weiler et al. 2022 [10]												14.5 ± 2.2°	6.8 ± 1.9°	N/A
Schneider, 2020 [16]	88% of patients were satisfied or very satisfied with the outcome and would recommend the procedure.		Mean at follow-up: 82 ± 14.1			5.6 ± 2.0	4.6 ± 1.7	4.2° ± 2.6° varus	0.8° ± 2.7° valgus		Mean score at follow-up: 71.8 ± 14.9			80% of patients returned to sport at some level. 31% returned to the same sport level as pre-injury. 17% returned to competitive sports.
Bonin, 2004 [15]										N/A	78.5 (46–100)			Fourteen patients (47%) returned to intensive sports, and 11 (37%) played moderate sports.

*IKDC* International Knee Documentation Committee.

**Table 3:** Complication.

Author, year	Complications
Badhe, 2002 [6]	One patient had an infection of postero-lateral corner ligament reconstruction, resulting in complete disruption of lateral tissues. One patient had non-union of open-wedge tibial osteotomy.
Jin, 2018 [11]	Three patients complained of hyperesthesia in the antero-lateral part of the proximal tibia and one patient had pain in the incision site. There was no case of non-union of the osteotomy site. No other major complications were noted.
Vaishya, 2016 [7]	There were no intraoperative complications. One patient (2.5%) had a loss of 20° of flexion. The average time for the radiological union of the osteotomy was 3.56 months (range 3–6 months), and two patients (5%) showed delayed union. No neurovascular complications were seen.
Mehl, 2016 [8]	No complications happened. This study shows that additional one-staged reconstruction of the ACL, however, seems to even improve knee function without significant risk of increased OA progression or increased complication rate in a mid-term perspective.
Sonnery-Cottet et al. 2014 [12]	Intra Operation: 0
	Post Operation: 0
	Graft failure: 0
Song et al. 2020 [13]	Intra Operation: 0
	Post Operation: 0
	Graft failure after ACLR: 0
Akoto et al. 2020 [14]	Intra Operation: 0
	Post Operation: 1 (5%): haematoma
	Graft failure after ACLR: 0
Rozinthe et al. 2022 [9]	Intra Operation: 0
	Post Operation: 0
	Graft failure after ACLR: 0
Weiler et al. 2022 [10]	Intra Operation: 0
	Post Operation: 1 (1.7%): implant infection
	Graft failure after ACLR: Not specified
Schneider, et al. 2020 [16]	8% failure rate, including one ACL graft rupture
Bonin, 2004 [15]	1 patella infera, 1 stiffness, 3 wound haematomas, 1 delayed wound healing, 7 DVT

## Result

**Figure 1:**
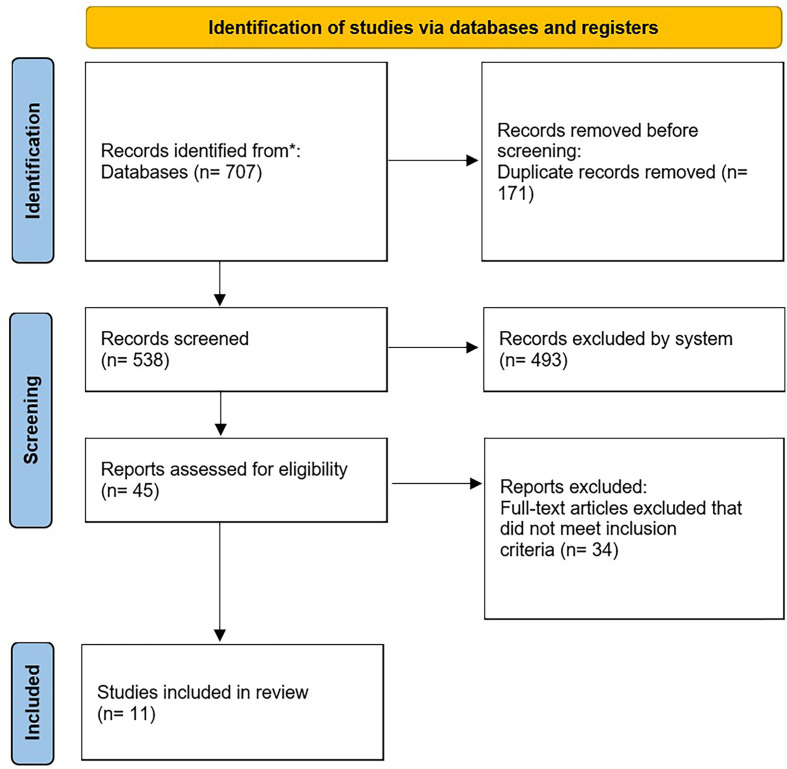
Preferred Reporting Items for Systematic Review and Meta-Analysis (PRISMA) flowchart of the article selection.

The systematic search performed using the previously mentioned keywords identified 536 studies after duplicates were removed. Of these, 493 were not related to our topic from title and abstract screening and some of those are review studies, leaving 43 studies. Of these, 34 studies did not include or report on instability, outcomes. After applying all exclusion criteria, 11 studies were considered for insightful data (Fig. 1).

**Figure 2:**
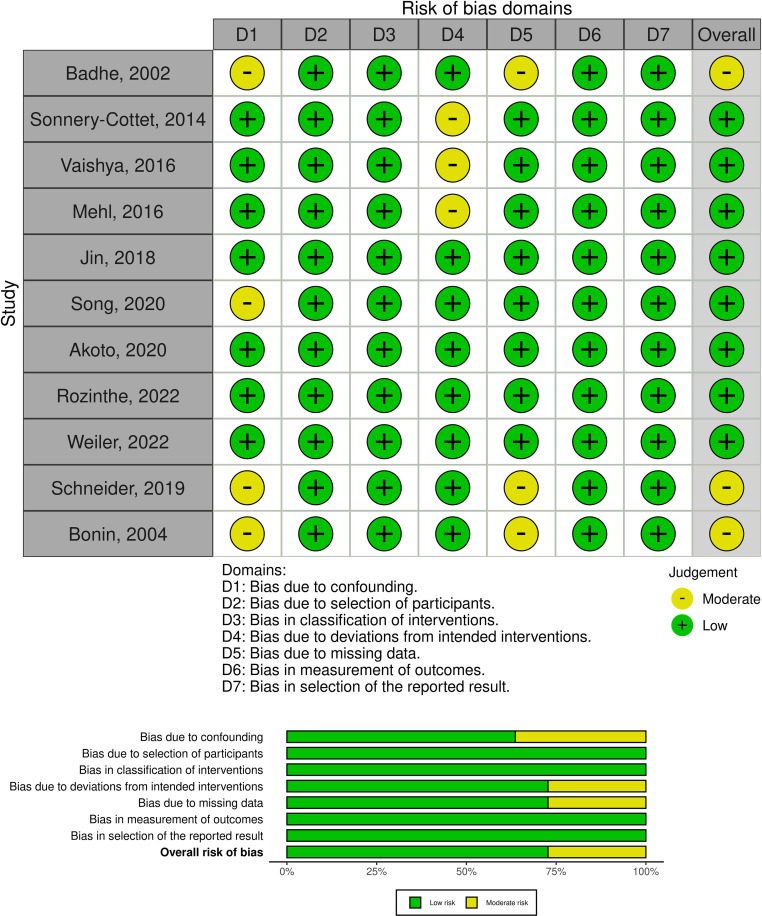
Risk of bias in non-randomised studies – of interventions.

Several included studies qualities were assessed and evaluated across seven domains of bias: confounding, selection of participants, classification of interventions, deviations from intended interventions, missing data, measurement of outcomes, and selection of the reported result. Each domain is color-coded, with green indicating a low risk of bias and yellow indicating a moderate risk. The overall bias is also assessed. The study by Badhe [6] shows a moderate risk of bias due to confounding, while Sonerry-Cottet [12] presents a moderate risk in deviations from intended interventions. Song [13] has a moderate risk of bias due to confounding. Despite these specific concerns, all other domains for these studies, as well as all domains for the remaining studies, are rated as low risk. Overall, the studies predominantly demonstrate a low risk of bias, indicating that their results are likely to be reliable (Fig. 2).

This review presents data from several studies examining the effectiveness of various surgical procedures in treating ligament instability. This study consistently shows that combining High Tibial Osteotomy (HTO) with Anterior Cruciate Ligament Reconstruction (ACLR) or Posterior Cruciate Ligament Reconstruction (PCLR) significantly reduces subjective instability. For instance, several studies [6, 8, 9, 13, 14], subjective instability was reduced from 100% preoperatively to 0% postoperatively.

Different combinations of procedures were used based on the specific ligament instability and patient condition. For example, Badhe [6] reported various combinations of ACL, PCL, and PLRI reconstructions, while Jin [11] focused on ACL and medial compartment osteoarthritis.

This review sheds light on the varied approaches and patient demographics reported in studies conducted across multiple countries. These studies, although differing in sample size, follow-up periods, and specific data points, offer valuable insights into patient outcomes in diverse healthcare environments. For instance, studies such as those by Jin [11] in South Korea and Mehl [8] in Germany provided detailed metrics on patient age and BMI, while others like Badhe [6] and Rozinthe [9] lacked specific information on these parameters. However, the overall trend reveals a predominance of male patients, with most studies involving a higher proportion of males than females, and the follow-up periods ranged from a few months to nearly a decade, highlighting both short-term and long-term research efforts [6, 8, 9, 11, 17].

The geographical diversity of these studies – spanning countries such as the United Kingdom, South Korea, India, Germany, France, and China – also underscores the global interest in the subject, contributing to a broader understanding of treatment outcomes and variations in healthcare practices. While some studies had relatively small sample sizes, others like Weiler et al. [10] in Germany offered larger patient populations, enhancing the statistical power of their findings.

This review illustrates the importance of standardized reporting across international studies, as the absence of key data points like BMI or patient age in some reports limits the potential for cross-comparative analysis.

Improvements in Lachman and Pivot Shift test grades were noted postoperatively, with significant improvements reported by Jin [11], while Bonin et al. [15] also mention considerable improvement in Lachman test. Both single-stage and two-stage procedures were effective, with Akoto et al. [14] highlighting the success of two-stage ACLR with HTO in revision cases. Additionally, the impact of detachment of the Anterior Tibial Tuberosity (ATT) and patellar tendon was explored, showing varied outcomes in pivot shift grades. Dejour et al. [18] provided early evidence that combining ACL reconstruction with valgus HTO yielded positive outcomes in terms of stability and delayed osteoarthritis. This aligns with findings from newer studies, confirming the procedure’s role in maintaining knee function over time [11, 15, 18].

Overall, the review underscores the effectiveness of combining HTO with ligament reconstruction in treating ligament instability, with significant improvements in both subjective and objective measures of knee stability.

This review presents data from various studies on knee surgeries, highlighting significant improvements in patient outcomes postoperatively. Satisfaction rates were high, with Mehl [8] reporting 92% of patients satisfied, and Jin [11] and Akoto et al. [14] providing satisfaction scores of 58.5 ± 12.0 and 49.9 ± 21, respectively. The Lysholm scores showed substantial increases across studies, such as Jin [11] reporting an improvement from 58.5 ± 12.0 pre-op to 94.0 ± 5.9 post-op, and Song et al. [13] showing an increase from 46.5 (34–58) pre-op to 89.5 (78–94) post-op.

The Tegner Activity Scores also improved, with Jin [11] noting an increase from 5.3 ± 0.9 pre-op to 7.0 ± 2.3 post-op, and Song et al. reporting an increase from 5.7 (4–6) pre-op to 7.3 (6–8) post-op. Mechanical axis alignment showed significant changes, with Jin reporting a shift from −1.2 ± 1.4 pre-op to 10.2 ± 2.3 post-op, and Song et al. showing a change from 18.5° (17°–20°) pre-op to 8.1° (7°–9°) post-op.

The Subjective IKDC Questionnaire scores improved notably, with Vaishya reporting a postoperative score of 87.5 (range 60–100), and Akoto et al. [14] showing an increase from 2.9 ± 1.5 pre-op to 6.1 ± 0.9 post-op. Posterior tibial slope measurements also showed positive changes, with Sonnery-Cottet et al. [12] reporting a decrease from 13.6° (13°–14°) pre-op to 9.2° (8°–10°) post-op, and Rozinthe et al. [9] showing a decrease from 13.2° ± 2.6° pre-op to 4.4° ± 2.3° post-op.

Return to sport and activities was high, with Badhe [6] reporting 93% of patients able to participate in light recreational activity post-op, and Vaishya [7] noting that 87.5% of patients participated in preoperative sports, 91% experienced a better quality of life, and 92.5% returned to the same occupation pre-op. Song et al. [13] reported a 100% return to sport/activities. Overall, the studies indicate significant improvements in satisfaction, functional scores, mechanical alignment, and return to activities post-surgery.

In the study by Badhe, one patient experienced an infection in the postero-lateral corner ligament reconstruction, leading to complete disruption of lateral tissues, and another patient had non-union of an open-wedge tibial osteotomy. Jin reported three patients with hyperesthesia in the antero-lateral part of the proximal tibia and one patient with pain at the incision site, but no cases of non-union of the osteotomy site or other major complications.

Vaishya noted no intraoperative complications, though one patient (2.5%) lost 20° of flexion, and two patients (5%) had delayed union of the osteotomy, with an average radiological union time of 3.56 months. Mehl reported no complications, suggesting that additional one-staged ACL reconstruction may improve knee function without increasing the risk of osteoarthritis progression or complications in the mid-term.

Sonnery-Cottet et al. [10] and Song et al. [11] both reported no intraoperative or postoperative complications, and no graft failures after ACL reconstruction. Akoto et al. [12] reported one postoperative complication (5%) involving a hematoma, but no graft failures. Rozinthe et al. [13] also reported no intraoperative or postoperative complications, and no graft failures. Weiler et al. [14] reported one postoperative complication (1.7%) involving an implant infection, but did not specify graft failure rates. Lastly, the earliest study conducted by Bonin et al. reported a few complications, such as: 1 case of patella infera, 1 case of stiffness, 3 cases of wound hematomas, 1 case of delayed wound healing, and 7 cases of DVT. The patella infera and one case of stiffness resulted poor outcomes despite of the surgical correction. The other eleven complications were manageable afterward.

Overall, the studies indicate a low incidence of complications, with most issues being minor and manageable.

## Discussion

The systematic review shows that the HTO procedure consistently delivers successful and reliable outcomes. According to the studies conducted by Catin et al. [17], four distinct situations have been identified in which high tibial osteotomy may be advantageous: anterior laxity with varus osteoarthritis, chronic anterior laxity in the setting of varus with lateral ligamentous laxity, chronic anterior laxity in the setting of a high tibial slope, and chronic posterior laxity or posterolateral corner injury. It effectively addresses knee instability, including injuries to the ACL, PCL, or PLRI ligaments, with all patients experiencing improved stability. Objective measures, such as the Lysholm score, CKRS, Tegner activity score, mechanical axis, IKDC, and tibial slope, demonstrated significant improvement. Subjective assessments, including instability, Lachman, and pivot shift tests, also improved. The procedure has a low complication rate, enabling most patients to return to their previous level of sports activity.

The strength of the study is the longer time of follow-up, as well as the consistency of the surgery, performed by the same experienced surgeon. Our research has addressed the limitations of a previous study conducted by Dean et al. [19]. They were unable to conclude whether high tibial osteotomy (HTO) is better performed alone or in stages. From the studies we’ve compiled, HTO and ligament reconstruction show better results when performed together. According to Badhe [6], patients undergoing a single-stage procedure experienced improved outcomes and better final CKRS results. Additionally, this study has its own limitations. Mostly, the studies are retrospective research, with a level 4 evidence. Many studies did not include a control group, and the patient characteristics were heterogeneous. Additionally, some studies measured PTS using radiographs, which are prone to errors. Further research is needed to provide a more accurate and comprehensive understanding.

Our review consistently demonstrated that HTO, especially when combined with anterior cruciate ligament reconstruction (ACLR) or posterior cruciate ligament reconstruction (PCLR), significantly reduces subjective and objective measures of knee instability. Several studies [6–8], showed a reduction in subjective instability from 100% preoperatively to 0% postoperatively. This consistent improvement highlights the reliability of HTO combined with ligament reconstruction in stabilizing the knee joint [20]. Similarly, Dean et al. [19] found that HTO improves knee stability across 13 studies, though complication rates varied widely. Stride et al. [21] also reported significant improvements in knee stability with combined HTO and ACLR, but noted complication rates ranging from 0% to 23.5%. These findings support the effectiveness of combined procedures but highlight variability in outcomes and the need for further research. The inclusion of long-term data from Schneider et al. [16] provides valuable insights into the sustainability of outcomes post-HTO combined with ACL reconstruction. This study demonstrated a high rate of return to sport (80%), although only 31% could resume their pre-injury level, and 17% returned to competitive levels. Notably, this aligns with other reports highlighting the challenges in maintaining high-level athletic performance after such procedures. Additionally, Schneider et al. [16] found a mean reduction in anterior tibial translation to 5.1 ± 3.8 mm, reinforcing the effectiveness of combined surgery for anterior laxity control. However, the 33% rate of osteoarthritis progression observed indicates that while stability can be achieved, long-term joint health remains a concern [17]. The long-term benefits of combining ACL reconstruction with HTO are supported by early evidence from Dejour et al. [18] who reported effective stabilization and pain management in patients with varus deformity and ACL laxity. This foundational work underscores the sustained value of combined procedures, corroborated by more recent studies like Schneider et al. [16] which found a 33% progression in medial compartment osteoarthritis. Such findings indicate that while the combined approach ensures stability, careful patient selection and counselling about potential long-term osteoarthritic changes remain essential.

Functional scores such as the Lysholm score, Tegner Activity Score, and the International Knee Documentation Committee (IKDC) scores showed significant improvements postoperatively. For instance, Jin et al. [11] reported an increase in the Lysholm score from 58.5 ± 12.0 preoperatively to 94.0 ± 5.9 postoperatively, while the Tegner Activity Score improved from 5.3 ± 0.9 to 7.0 ± 2.3. These improvements indicate not only restored knee stability but also enhanced functional capacity and patient satisfaction. In comparison, Ollivier et al. [22] reported significant improvements in functional scores following high tibial osteotomy (HTO), with survival rates ranging from 86% to 100% at 5 years, 64% to 97.6% at 10 years, 44% to 93.2% at 15 years, and 46% to 85.1% at 20 years. Similarly, Oberg et al. [23] found statistically significant improvements in 6 out of 20 functional variables after 6 months and in 10 out of 20 variables after 12 months, although the treatment goal was not reached for most variables, suggesting that postoperative training might have been inadequate. Additionally, van Wulfften et al. [24] reported survival rates of 75–94% at 5 years, 51–95% at 10 years, and 30–90% at 15 years, highlighting reductions in pain and improvements in function post-HTO. Collectively, these studies underscore the positive impact of HTO on functional outcomes, though the extent of improvement and long-term success rates vary.

Radiographic outcomes such as the mechanical axis alignment and posterior tibial slope also showed substantial improvements. Jin et al. [11] reported a shift in the mechanical axis from −1.2 ± 1.4 preoperatively to 10.2 ± 2.3 postoperatively, reflecting the effectiveness of HTO in realigning the knee joint. Similarly, the posterior tibial slope improved significantly in studies by Sonnery-Cottet et al. [12] and Rozinthe et al. [9], further supporting the role of HTO in correcting knee deformities and enhancing stability. Studies comparing high tibial osteotomy (HTO) techniques, such as those by Higuchi et al. [25] and Li et al. [26], show significant improvements in mechanical axis alignment, with OWHTO being particularly effective in correcting varus deformities. The studies by Higuchi et al. [25] emphasize that achieving a postoperative valgus angulation of 8°–10° enhances joint stability and alignment. These findings align with the improvements reported in some studies [9, 11, 12], underscoring HTO’s effectiveness in improving both mechanical axis alignment and posterior tibial slope.

This review identified a low incidence of complications associated with HTO. Most complications were minor and manageable, such as hyperesthesia and pain at the incision site reported by Jin et al. [11], and a hematoma reported by Akoto et al. [14]. The overall safety profile of HTO, combined with its efficacy in improving knee stability, makes it a viable option for treating knee instability. Similarly, Li et al. [26] highlights that postoperative complications can include infection, loss of correction, and non-union, with a complication rate of 10–15%. These findings align with the review’s identification of minor and manageable complications, such as hyperesthesia, pain at the incision site, and hematoma, underscoring the overall safety profile of HTO.

The long-term effectiveness of HTO combined with ligament reconstruction was evidenced by studies with extended follow-up periods. Two studies [8, 9] demonstrated sustained improvements in knee stability and functional outcomes over several years, indicating the durability of these interventions. A systematic Review by He et al. [27] found that high tibial osteotomy (HTO) has a survival rate of 74–80% at ten years, which decreases to 57–67% by 15 years, indicating good long-term effectiveness. These findings align with the long-term effectiveness reported by previous studies [8, 9], who observed sustained improvements in knee stability and functional outcomes over several years.

By this time, HTO has proven to be an effective treatment for both acute and chronic ACL instability, as well as PCL and posterolateral corner (PLC) laxities. In a study by Badhe et al. [6], 14 patients with varus-angulated ACL/PLC ligament-deficient knees were treated: five underwent ACL reconstruction, six received LARS posterolateral ligament reconstruction with a tibial osteotomy, and three had a tibial osteotomy without ligament reconstruction. Twelve knees (86%) remained stable, enabling patients to engage in leisure sports. This study suggests that if posterolateral structures are lax but not completely disrupted, an opening wedge tibial osteotomy without ligament reconstruction can stabilize the knee, potentially eliminating the need for ligament reconstruction. But, the effectiveness of HTO in cases of chronic instability, with or without ligament reconstruction, is debated. According to Kim et al. [28], patients with PCL or ACL instability exhibit inferior knee stability when only HTO is performed compared to those who receive combined HTO and ligament reconstruction. Despite this, all patients show significant improvement in knee stability compared to their preoperative condition.

In ACL-deficient knees, increased anterior tibial translation during weight-bearing activities accelerates osteoarthritis progression. This translation is influenced by several factors, including the ACL, the posterior horn of the medial meniscus, the posteromedial capsule, and the posterior slope of the tibia. Increased posterior tibial slope (PTS) is associated with greater anterior tibial translation (ATT) in ACL-deficient knees. Specifically, studies indicate that every 10° increase in PTS can lead to a 6 mm increase in passive anterior tibial translation, contributing to higher strain on the ACL [29]. A study by Bernhardson et al. [29] investigated the relationship between sagittal plane posterior tibial slope and PCL (posterior cruciate ligament) injuries. The main finding was that a decreased posterior tibial slope is associated with PCL tears compared to cruciate ligament-intact controls. Most PCL injuries occurred due to contact mechanisms involving posteriorly directed force to the proximal tibia. Patients with noncontact PCL injuries had significantly reduced posterior tibial slope. A flattened tibial slope of less than 6° may increase PCL injury risk. While increased posterior tibial slope is protective for PCL-deficient knees, the impact of decreased slope on tibial sag or position remains unexamined. Further research is needed to validate these clinical correlations, especially in PCL-reconstructed knees.

In other studies [10, 13], anterior close wedge high tibial osteotomy during primary ACL reconstruction was performed on young, active patients with significant anterior instability and increased PTS, believing that ACL reconstruction alone would not sufficiently restore knee biomechanics and stability. They also highlighted that, when performed by experienced surgeons, anterior close wedge high tibial osteotomy is an effective procedure with a low risk of complications to restore stability and protect the ACL reconstruction failure.

This systematic review is subject to several limitations. First, as with many reviews, there is a potential for publication bias since we rely heavily on previously published studies, which may over-represent positive outcomes and under-report negative or null results. Additionally, the follow-up data in many of the included studies is limited, both in terms of duration and consistency, which may impact the generalizability and long-term applicability of our findings. These factors should be considered when interpreting the results of this review. Various technologies have been introduced into HTO practice, primarily to enhance the accuracy and precision of correction angles during high tibial osteotomy [3, 30]. More research is needed on these surgical assistance technologies, such as HTO planning software and computer-assisted navigation. However, only a limited number of studies have been conducted, and it appears that technological assistance does not significantly improve outcomes compared to conventional methods.

## Conclusion

Based on our comprehensive analysis of data from 11 studies involving 303 patients, HTO for knee instability caused by ACL or PCL deficiency shows improved midterm and long-term outcomes, and patient-reported postoperative satisfaction compared to postoperative conditions. Only a few complications have been reported in this procedure, usually due to external factors and not directly related to the surgical technique. Currently, HTO stands out as the most effective treatment option in the majority of cases.

## Data Availability

The data supporting the findings of this study can be obtained from the corresponding author upon reasonable request.
